# Occupational Health Injuries by Job Characteristics and Working Environment among Street Cleaners in South Korea

**DOI:** 10.3390/ijerph17072322

**Published:** 2020-03-30

**Authors:** Jungmin Park, Junse Lee, Myung-Sun Lee

**Affiliations:** 1 School of Nursing, Hanyang University, Seoul 04763, Korea; 2 System LSI Business Department, Samsung Electronics, Hwaseong 18448, Korea; junselee@utexas.edu; 3 Health Convergence, Ewha Womans University, Seoul 03760, Korea

**Keywords:** accident, incident, injury, street cleaners, waste collector

## Abstract

In 2018, 1822 incidents relating to death or injury occurred among street cleaners in South Korea. However, South Korea currently lacks comprehensive studies on related injuries based on street cleaners’ job characteristics and environments in the country. This study analyzed injuries according to the job characteristics and environment through a survey of 150 Korean street cleaners working in the Seoul and Gyeonggi-do areas. This study assessed three category measures—demographic, job characteristics, and environments—to determine the effects of injuries. The demographic measures consisted of age, gender, and education level. Job characteristic variables consisted of length of time on the job, job contract, monthly income, working hours per day, working start time, overtime per month, and days off per month. For job environments, this survey included job duty, classification, main tasks, work intensity, and safety equipment. The data were analyzed according to descriptive statistics, injury ratio, and Probit regression analysis. The results of the analysis demonstrated that the participants with the highest risk of injury were mostly males with less than a middle school education. Assessment of the job characteristics showed that the most prevalent length of working experience was less than 5 years, with most engaging in contract/day work. A share of 36.67% of the participants reported injuries. The most prevalent reason for injury was overwork (32.73%), and the most frequent injury area was the lower back (49.09%). In summary, injuries among street cleaners were associated with education level, job experience, days off from work, and work intensity. As such, street cleaners should receive more education to decrease the risk of injuries, regardless of the number of employees or their contract status.

## 1. Introduction

In South Korea, 971 workers died in 2018 due to workplace incidents, and 2.7 workers die due to injuries in industrial settings, on average, each day [1]. Of occupations with high fatal work injury rates in 2018, street cleaners ranked fifth in the dangerous job ranking in the United States [2]. Street cleaners perform jobs that are essential to society because they help maintain health and hygiene in our communities [3]. The work of street cleaners in South Korea includes a wide range of job duties in diverse work environments, such as collecting, loading into trucks, and incinerating garbage bags of refuse or recyclable materials [3,4,5,6]. In South Korea, the public sector usually hires street cleaners [7,8] who clean streets and collect garbage from the roads using dustpans, brooms, and plastic garbage bags [3]. Nevertheless, not all street cleaners enter contracts with the government (18,992 people); some also contract with private cleaning businesses (24,398 people) [3,9]. In a previous study, most fatal work injuries consisted of transportation accidents, falls/slips/trips, violence/other injuries by person or animal, contact with objects/equipment, and exposure to harmful substances or environments [1]. As a result, street cleaners’ health is often put at risk due to the dangerous nature of their jobs [8], such as collecting hazardous aerosols and liquids, exposure to infections and sharp objects [10], and inhalation of dust and biological hazards during waste collection [11]. 

A total of 43,390 street cleaners worked in South Korea in 2018 [12]. The majority were over 50 years old and accounted for 61.6% of street cleaners (n = 1123). The majority of incidents occurred during early morning or at night, and the work duty consisted of collecting or moving waste that was usually allocated to a private contract business [12]. The areas with the most injuries were Gyeonggi-do (n = 341, 18.7%) and Seoul (n = 329, 18%) (N = 1822) because most street cleaners work in these two cities; these are also the major cities in South Korea [12]. Usually, the main reasons for incidents were related, in that all of these tasks corresponded with the occupational health problems that street cleaners experienced during these activities, including frequent use of joints such as their knees, shoulders, elbows, ankles, and necks, as well as their backs when they collected waste, loaded it, and drove trucks during collection [3]. 

The incident count among street cleaners was 1822 over 3 years (2015–2017); 18 people died, and 1804 sustained injuries [12]. The most common cause of those deaths was car accident (n = 9, 50%) and strain (n = 6, 31%) (N = 18) [12]. The main causes of injuries were slipping (n = 353, 19.4%), falling (n = 293, 16.1%), and brain or cardiovascular disease (n = 279, 15.3%) (N = 1804) [12]. The major injury factors among street cleaners in South Korea in 2016 were cuts from sharp objects (49.2%), moving heavy garbage bags (28.8%), slipping (26.8%), and falling (13.9%) [12]. It is clear from previous studies [3,5,11,13,14,15] that street cleaners have more exposure to injury. Despite such awareness, South Korea lacks comprehensive studies on the risk factors of work-related injury in street cleaners’ job characteristics and environments. Moreover, most existing studies in other countries focused on musculoskeletal disease regarding overuse of joints while collecting waste, or on diseases [3,5,11,13,14,15]. Despite this, injuries relate to street cleaners’ job characteristics and working environment, so more comprehensive studies must be undertaken to discern the relationships between those variables. To reduce the gap, our study focused on job characteristics such as working duties, so it will prove useful in preventing injuries. This study sought to analyze the job characteristics (length of time on job, job contract, monthly income, working hours, overtime, days off) and environment (duty, classification, main tasks, work intensity) of street cleaners’ job-related injuries in South Korea. With these specific aims, the authors will determine the effect factors of occupational health injuries by job characteristics and working environments among street cleaners in South Korea.

## 2. Materials and Methods 

### 2.1. Study Design and Conceptual Model

**Figure 1:**
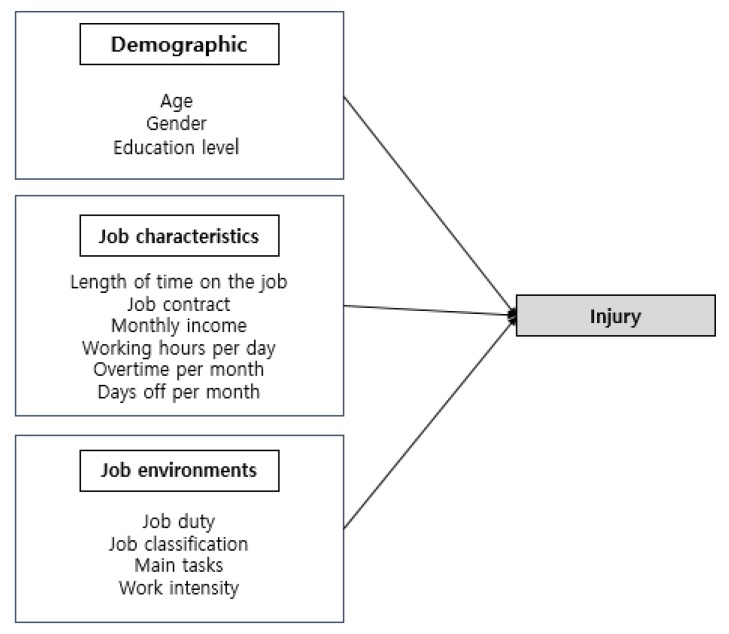
Conceptual model of occupational injuries.

This research conceptual model was based on Lim’s (2010) study, which focused on the relationships between injury and job environments such as job duty, tasks, and intensity [16]. For this study, the authors added survey questions related to job characteristics (job contract, income, working hours, overtime, days off) because it is important to understand relationships between these variables and job injury rates, since the majority of South Korean street cleaners are currently contract workers, so this might affect injury reporting. Guided by previous research, the authors created a conceptual model based on a hypothesis (Figure 1). This cross-sectional descriptive study analyzed four variables: demographics; job characteristics; job environments; and injuries (Figure 1). This study used a self-report, paper–pencil survey. Standard survey question methods for South Korean street cleaners’ job characteristics and injuries have yet to be developed. Thus, the authors conducted a pilot study (N = 30) (Cronbach α = 0.7). This study included randomly selected street cleaner workers in the public sector in the cities of Seoul and Gyeonggi-do, South Korea. The researchers identified public sector and private contract cleaning businesses in Seoul and Gyeonggi-do and then contacted them to explain the research purpose, after which they visited sites during street cleaners’ daytime working hours. The data were collected by self-reporting in 2013 from 10 public sector employers. The participants were over 18 years of age, could read and write Korean, worked as street cleaners in the public sector, and were willing to participate in this study. We conducted a G-power analysis to calculate the sample size [17,18]. At least 75 participants are needed provide representative results with medium effect size (α = 0.07). A total of 150 participants were included in this study. The Ewha Womans University institutional review board (IRB) in Seoul, South Korea, approved this study (IRB number: 62-7).

### 2.2. Variables and Data Analysis

To analyze injuries among street cleaners, this study determined the descriptive statistics and injury ratio according to demographics, job characteristics, job duty, and injury site using IBM SPSS Statistics for Windows, 22.0 [19]. This study defined the statistical significance as *p* < 0.10. These were presented as the frequencies, percentages, and injury ratios. 

The demographic variables consisted of age (three categories: ≤30, <30–≤50, and over 51 years of age), gender (two categories: male and female), and education level (two categories: below/above high school) (Table 1). The job characteristics consisted of the length of time on the job, job contract, monthly income, working hours per day, working start time, overtime per month, and days off per month. The length of time on the job consisted of five categories based on the number of years (<1, 1–<3, ≤3–<5, ≤5–<10, and ≥10 years). The job contract included three categories (regular, contract, and day worker). The government of South Korea hires regular workers, who commonly receive benefits, such as health insurance, pensions, and higher salaries than those of contract and day workers. Regular workers are generally guaranteed to work until 65 years of age, which is the official retirement age in South Korea. Contract workers are also hired by the Korean government and usually receive health insurance, but not pensions. They generally need to renew their employment contracts every 1–2 years. Day workers are not hired by the Korean government and typically do not receive health insurance or pension benefits. They are hired temporarily and as needed. Monthly income was calculated by converting the South Korea won (₩) to U.S. dollars (₩1000 = $1). Monthly income was categorized according to income range (two categories: ≤$2000; and ≥$2000). Working hours per day were separated into two categories: no more than 8 hours and more than 9 hours per day. Working start time was a dichotomous variable (0: regular, 1: irregular (shift work)). Overtime and days off per month variables were also determined. 

For the analysis of a street cleaner’s job environments, the present study divided the job environments into job duty, classification, main tasks, work intensity, and safety equipment. The job duties included seven categories (can be multiple choices), namely, street cleaning, waste collection/carriage, recycling collection, waste landfill/incineration, sewage/human waste removal/disposal services, and factory/building cleaning. Street cleaning was defined as manual street sweeping or collection of refuse using a broom and/or handcraft. Waste collection and carriage consisted of collecting waste from streets, apartments, houses, and buildings, including pulling a handcart and manually handling waste bags. Recycling collection was defined as the collection of only recyclable waste from streets, apartments, houses, and buildings, and included collecting bulky and heavy waste (i.e., furniture, home appliances, etc.). Waste landfill and incineration was defined as placing waste from all areas in a landfill for incineration. Sewage/human waste and disposal services included disposing of waste from humans and animals. Factory/building cleaning included cleaning and collection of waste from factories or buildings. The job classifications were further divided into street cleaner, truck driver during collection, garbage collector, recycle waste collector, and others. The main job tasks were also divided into five categories (can be multiple choices), namely, sweeping/collecting waste into garbage bags, loading of waste (lifting waste bags or pulling at height operating machinery), moving garbage bags, waste sorting (garbage separation collection site), and others. Work intensity was defined according to two measurements, namely, the number of garbage bags and work intensity. The garbage bags were 100 L, the largest size available in South Korea. Each street cleaner was categorized as a regular, contract, or day worker. Furthermore, each street cleaner had different working times per day, so the work intensity differed by duty hours (≤10 bags, 11–30 bags, and ≥31 bags). Thus, the number of garbage bags was used to determine the work intensity. This study used a self-reported, 5-point Likert scale for workers to indicate their work intensity as very difficult, often difficult, average difficulty, seldom difficult, or never difficult. For safety equipment, the government recommends that street cleaners wear hard hats while performing their job duties. The participants self-reported their compliance using a 5-point Likert scale of always, often, average, seldom, and never wear.

The injury sites consisted of the lower back, neck/shoulder, arms/elbow, hands/finger, foot, and other. The injury risk factors consisted of overwork, occupational diseases, slip, motor vehicle accident-related, crush, strain, fall, and other. Occupations at the time of injury were divided into recycling and large waste (over 20 kg) collection, street cleaning, household waste/food waste collection, truck driving during collection, and other.

To determine the injuries based on job characteristics and environments among street cleaners—except for multiple choices such as job duty, classification, and main tasks—this study used Probit regression analysis using the STATA version 13 program [20]. Probit regression is a useful method to analyze limited dichotomous independent variables, which in this case consisted of injured and not injured. Two categories were defined for the Probit regression analysis (0: not injured; 1: injured). Using this, positive and negative Probit regression results indicated that the variable increased or decreased injury occurrence rates, respectively.

The dependent continuous variables were age, job experience (in years), days off, and job intensity. The dependent categorical variables were education level, gender, job contract, working hours, and safety equipment.

## 3. Results

Previous studies focused on symptoms of diseases, such as musculoskeletal disease. Here, however, we needed to realize which factors will affect the rate of injury among street cleaners and how injury in working environments can be prevented. This research aimed to identify relationships between possible effect factors of injury and job characteristics/environments.

### 3.1. Study Characteristics and Injuries

A total of 150 participants were included in the analysis. The most prevalent age groups were < 30–≤50 years (73.33%). Those aged more than 51 years and less than ≤30 years accounted for 20% and 6.67% of the study population, respectively. Most street cleaners were male (86%), and the highest education level was high school (46%) (Table 2). Of all of the participants (N = 150), 55 had experienced injuries. The characteristics with the highest injuries were age <30–≤50 years (injured n = 44), male gender (injured n = 48), and below high school education level (injured n = 33).

### 3.2. Street Cleaners’ Job Characteristics and Injuries

Most study participants responded that they had held their jobs for 3–<5 years (28%), followed by 5–<10 years (21.33%), 1–2.9 years (20.67%), less than 1 year (16%), and more than 10 years (14%). More than half of the participants were contract workers (58%). Only 24% were regular workers, leaving around 76% who were not regular workers. The most prevalent monthly income range was ≤$2000 (78.67%) per month. Based on salary, those with the highest injury earned ≤$2000 per month (injured n = 43).

The present study also investigated the participants’ length of working time and working start time. More than half of the street cleaners reported that they usually worked 9 to 10 hours per day (57.33%) and had irregular start times (shift work) (58%). In addition, 90.67% of the street cleaners worked no fewer than 9 hours per day. Further, the street cleaners worked an average overtime of 9.13 ± 1.50 hours per month, and they had only 4.2 ± 8.39 days off during the month. We conducted a chi-squared t-test to learn the differences between groups. Only length of time on job (years) and days off were significant, *p* < 0.10. Length of time on job (years) and days off per month (days) were significantly different between the injured and not injured groups: t = 2.277, *p* < 0.05 and t = 1.825, *p* < 0.10, respectively.

### 3.3. Street Cleaners’ Job Environments and Injuries

The most prevalent job duties were waste collection/carriage (32.67%), street cleaning (31.33%), and recycle collection (19.33%) (N = 150) (Table 3). The most frequent job classification was street cleaner (33.33%). The main tasks of more than 40% of the participants consisted of sweeping/collecting (43.33%) and loading (lifting/carrying) garbage bags (32%). 

The highest number of injuries occurred during waste collection/carriage (injured n = 18). By job classification, the jobs with the highest number of injuries were truck driving during collection (injured n = 16) and street cleaning (injured n = 15). Among those who were injured, the injuries occurred based on their main tasks was highest for waste loading (lifting/carrying garbage bags) (injured n = 25), followed by sweeping/collecting garbage bags (injured n = 20).

Regarding job intensity, 44% of the participants (N = 150) disposed of more than 31 (100 L) garbage bags per day, corresponding to handling 3100 L of garbage during duty. More than half of the participants described their work as often difficult (55.33%), while 20% reported it to be very difficult. Also, a significant difference appeared between injured and not injured in job intensity (t = 2.277, *p* < 0.10).

The highest injury rate according to work intensity was among those who handled more than 31 (100 L) garbage bags per day, with injuries reported by 32 workers. This finding indicated that disposing of more garbage bags resulted in more injuries. The self-reported work intensity revealed that the work was more often categorized as difficult among workers who sustained injuries, which, in turn, resulted in the highest injuries (injured n = 34).

Most street cleaners wore the only suggested safety instrument, a hard hat; only 8% (n = 12) did not wear one while working. Nonetheless, the results showed the opposite in injuries; even though they overwhelmingly wore the hard hats, they still suffered injuries.

### 3.4. Street Cleaners’ Injury Sites, Risk Factors, and Occupation at the Time of Injury 

The most prevalent injured body location was the lower back (49.09%) (N = 150) (Table 4). The major injury risk factors were overwork (32.73%), followed by occupational disease (30.91%), slipping (9.09%), and motor vehicle accident and crush injury (7.27%). The primary occupations at the time of injury were collecting recycle/large waste (over 20 kg) (49.09%), street cleaning (25.45%), and collecting household waste/food (14.55%). 

### 3.5. Analysis of the Determinants of Injury in Street Cleaners

The analysis showed a lower injury rate among those with a higher level of education (*p* < 0.10) (Table 5). Age and gender, however, were not significant determinants of injury occurrence in street cleaners. Based on job experience, participants with 3–<5 years’ work experience (*p* < 0.10) and those with more than 10 years’ work experience (*p* < 0.05) had more injuries than workers with no more than 1 year of job experience. On the contrary, having fewer than 3 or 5–<10 years of job experience had no significant effect on injuries. Job contract and working hours did not significantly affect the occurrence of injuries, but having more days off was associated with an increased injury rate (*p* < 0.05). This result could be caused by workers’ job contracts, since most respondents worked as contract/day workers and likely had more days off than regular workers. Finally, having a difficult job with high intensity also increased the number of injuries (*p* < 0.10).

## 4. Discussion

The street cleaners included in this study had a high number of injuries based on the job characteristics and environments. More than one-third of the participants had previously sustained injuries related to on-the-job characteristics and environment. This study revealed several key findings. Based on the length of time on the job, those who had worked fewer than 5 years as street cleaners and those who worked more than 9 hours per day sustained the highest number of injuries. In South Korea, 30% of street cleaners have been working fewer than 5 years and more than 40% of workers work more than 8 hours per day [12]. Based on our research results, the explanation for these findings could be that participants with less experience had a higher risk of injury due to inexperience, while longer working hours combined with heavy workloads might have increased the risk of injuries.

Additionally, the most prevalent injuries occurred in the lower back and legs/neck/shoulder, which were related to the tasks of lifting and carrying garbage bags. These injuries were related to the workers’ musculoskeletal strength. Continuous stress on the musculoskeletal system due to performing actions such as lifting, pushing, and pulling heavy loads can often result in musculoskeletal problems [11,15,21,22,23]. Due to the heavy workload, 75.33% of participants reported experiencing difficult work intensity more than often, and 44% reported moving more than 31 (100 L) garbage bags while on duty—findings similar to those of a previous study [9]. In South Korea, street cleaners reported lifting 7765.2 kg per day on average [12]. The previous study also indicated that waste collection (42.8%) and loading of waste disposal materials (29.5%) caused the most injuries while street cleaners worked (N = 325) [9]. This could lead to pain in the lower back caused by lifting heavy garbage bags [9,24] due to poor or incorrect posture [9]. Such a posture can cause lower back pain. Because of this, changes are needed to reduce the workload per duty or decrease the garbage bags’ weight to prevent injuries.

Aspects of determinants of injuries, education level, and job experience, as well as more days off and greater job intensity, translate into an increased risk of injuries. One study suggested that employers with more than 50 employees should provide safety education to prevent occupational injuries [7]. Interestingly, safety equipment were not determinants of injuries in the present study. The helmets were not a determinant because the injuries occurred in places on the body other than the head. The street cleaners could not have had appropriate equipment or education regarding their use, or workers might wear only hard hats for safety. Furthermore, it could be possible that workers had not received proper training because companies in South Korea with fewer than 50 employees usually do not have health and safety protocols [9]. Therefore, street cleaners should undergo additional training to decrease their risk of injuries regardless of the number of employees in a business or their contract status, and require more safety equipment. 

According to the research results, it is necessary to reduce injuries. In South Korea, no government standard safety education manual exists, so it is difficult to provide standard education and manage training quality. Seeing this, it would prove beneficial to develop a government-endorsed safety education manual to prevent injuries.

In addition, most countries, including the United States, conduct regular injury surveys of street cleaners so as to identify the dangers related to the working conditions of street cleaners and the efforts that can be made to prevent injuries [2]. However, in South Korea no such regular surveys are conducted, and there is no standard tool for surveying. It might be helpful to rank injuries by job and the factors necessary to prevent injuries. Finally, street cleaners in South Korea use regular garbage bags. From the results reported, street cleaners’ primary reasons for injuries consisted of collecting waste and loading it [12]. It might be helpful to limit the bag size street cleaners can lift per day or the size of the bag and, through this action, musculoskeletal injuries might be reduced, which concurs with previous results’ suggestions [9,11,15,21,22,23,24].

Even though this study took place using appropriate statistics, some limitations exist. This one-time survey study was limited to two large cities in South Korea; there is thus a risk of generalization bias. What is more, this study used a self-reporting survey in which the researcher assumed that the answers were accurate and honest. Likewise, no standard survey tool can determine job characteristics and environments for street cleaners’ injuries. Consequently, future research should define a standard survey tool and be conducted with a large sample in a large area, not just in big cities. Despite these limitations, this study proves significant because its findings allow healthcare professionals to provide guidelines that can decrease and prevent injuries based on job characteristics and environments. 

## 5. Conclusions

This study provided an overview of the injuries based on job characteristics and environments among street cleaners in South Korea. Most street cleaners had low educational levels, worked as contract/dayworkers, and worked overtime. During waste collection, they had an increased risk of sustaining injuries, which were the primary causes of musculoskeletal disorders. To better prevent such injuries, health care professionals need to insist that employers reduce workloads and provide appropriate training regarding workplace safety.

### 5.1. Applying Research to Practice

Occupational health nurses need to educate those who are at risk of injury in their work environments. To promote health and reduce occupational injuries, it is important for occupational health nurses to educate workers regarding injury prevention. Providing care to street cleaners is especially important because they work in dangerous environments due to their job characteristics and major risk factors related to their education levels. Thus, as educators in the role of occupational health nurses, providing methods for injury prevention is important.

### 5.2. Implications of Occupational Safety and Health Professionals

Industrial work injured 89,848 people in 2017 in South Korea [25]. Most serious injuries were related to transportation accidents, fall, slips, trips, or violence [25]. The majority of these injuries could have been prevented through education. Even though we have guidelines to prevent injuries in each area, no regular training system is in place in South Korea. More specifically, we can prevent injuries for street cleaners based on job characteristics and environments. Following our research, by reducing workloads and providing appropriate training while considering job characteristics and environments, it could be possible to prevent many future injuries. Hence, health professionals must understand street cleaners’ job characteristics and provide safety training in the future.

### 5.3. Implications for Occupational Health Nurses

Occupational health nurses must advocate for reduced injuries based on job characteristics. The role of occupational health nurses here is to improve street cleaners’ health and prevent injuries. To reduce occupational injuries associated with lower educational level and overtime work, occupational health nurses should advocate better training for street cleaners at risk of occupational injuries.

## Figures and Tables

**Table 1:** Street cleaner characteristics and injuries variables.

Variables Category	Variables	Data	Number of Variables	Variables
Demographic	Age	Categorical data	3	≤30, <30–≤50, ≥51
	gender		2	male, female
	Education level		2	below/above high school
Job characteristics	Length of time on job(years)	Categorical data	5	<1, 1–<3, ≤3–<5, ≤5–<10, ≥10
	Job contract		3	regular, contract, day worker
	Monthly income		2	≤2000, >2000
	Working hours/day		2	≤8, >8
	Overtime/month(hours)	Continuous data	n/a	
	Day off/month(days)			
Job environments	Job duty	Categorical data	7	street cleaning, waste collection/carriage, recycle collection, e landfill/incineration removal/disposal, factory/building cleaning
	Job classification		6	street cleaner, truck driver for waste collection, garbage collector, recycling collector, others
	Job main tasks		7	Sweeping/collecting, loading, moving, waste arrangement, others
	Job work intensity		3	number of garbage bags/duty, work intensity
	Safety equipment		5	5-point Likert scale
Injury	Injury site	Categorical data	7	lower back, legs, neck/should, arms/elbow, hands/fingers, foot, others
	Injury risk factors		8	Overwork, occupational diseases, slip, motor vehicle accident related, crush, strain, fall, others
	Occupation at the time of injury		5	recycling/large waste, street cleaning, household waste/food waste, truck driving

**Table 2:** Street cleaner characteristics and injuries (N = 150)

Variables	Description	Frequency (*n*)	Percentage (%)	Injured (*n*)	Not Injured (*n*)
Age (years)	≤30	10	6.67	3	7
	<30–≤50	110	73.33	44	66
	≥ 51	30	20.00	8	22
Gender	Male	129	86.00	48	81
	Female	21	14.00	7	41
Education level	Below high school	73	48.67	33	40
	Above high school	77	51.33	22	55
Length of time on job (years)	<1	24	16	6	18
	1–< 3	31	20.67	11	20
	3–< 5	42	28	16	26
	5–<10	32	21.33	12	20
	≥10	21	14	10	11
Job contract	Regular worker	36	24	15	21
	Contract worker	87	58	33	54
	Day worker	27	18	7	20
Monthly income (USD)	≤2000	118	78.67	43	75
	>2000	32	21.33	12	20
Working hours per day	≤8	14	9.33	4	10
	>9	136	90.67	51	85
Overtime per month (hours)		9.13 ± 1.50 (average ± SD)			
Days off per month (days)		4.2 ± 8.39 (average ± SD)			

**Table 3:** Street cleaner job environments (N = 150).

Variables		Classification	Frequency (n)	Percentage (%)	Injured (n)	Not Injured (n)
Job duty		Street cleaning	47	31.33	13	34
		Waste collection/carriage	49	32.67	18	31
		Recycle collection	29	19.33	10	19
		Waste landfill/incineration	9	6.00	6	3
		Sewage and human waste Removal/disposal services	5	3.33	2	3
		Factory/building cleaning	11	7.33	6	4
Job classification		Street cleaner	50	33.33	15	35
		Truck driver for waste collection	36	24.00	16	20
		Garbage collector	14	9.33	5	9
		Recycling collector	32	21.33	10	22
		Others	18	12.00	9	9
Job main tasks		Sweeping/collecting garbage bags	65	43.33	20	45
		Loading (lifting/carrying) garbage bags	48	32.00	25	23
		Moving garbage bags	9	6.00	3	6
		Waste arrangement	15	10.00	4	11
		Others	13	8.67	3	10
Job intensity	Number of garbage bags (100 L)/duty	≤10 bags	33	22	6	27
		<10–≤30 bags	51	34	17	34
		≥31 bags	66	44.00	32	34
	Work intensity (5-point Likert scale)	Very difficult	30	20.00	21	9
		Often difficult	83	55.33	22	61
		Average difficult	31	20.67	9	22
		Seldom difficult	4	2.67	2	2
		Never difficult	2	1.33	1	1
Safety equipment (5-point Likert scale)		Always wear	73	48.67	25	48
		Often wear	45	30.00	21	24
		Average wear	20	13.33	4	16
		Seldom wear	6	4.00	2	4
		Never wear	6	4.00	3	3

**Table 4:** Injury sites, risk factors, and occupation at the time of injury (N = 55).

Variables	Descriptions	Frequency (*n*)	Percentage (%)
Injury site	Lower back	27	49.09
	Legs	8	14.55
	Neck/shoulder	7	12.73
	Arms/elbow	5	9.09
	Hands/finger	3	5.45
	Foot	3	5.45
	Others	2	3.64
Injury risk factors	Overwork	18	32.73
	Occupational diseases	17	30.91
	Slip	5	9.09
	Motor vehicle accident-related	4	7.27
	Crush	4	7.27
	Strain	2	3.64
	Fall	3	5.46
	Others	2	3.64
Occupation at the time of injury	Recycling/large waste (over 20 kg) collection	27	49.09
	Street cleaning	14	25.45
	Household waste/food waste collection	8	14.55
	Truck driving for waste collection	3	5.45
	Others	3	5.45

**Table 5:** Analysis of injury determinants by Probit analysis (N = 150).

Study Characteristics	Study Characteristics Explanation	Probit
Education level	Below high school Above high school	−0.0959 * (0.0568)
Age	Age	−0.0240 (0.0157)
Gender	Male, Female	0.361 (0.395)
Job experience (years)	1–<3	0.410 (0.389)
	3–<5	0.681 * (0.384)
	5–<10	0.615 (0.404)
	≥10	0.888 ** (0.452)
Job contract	Contract worker	−0.0425 (0.306)
	Day worker	−0.558 (0.410)
Working hours	≤8	−0.157 (0.401)
	>9	0.288 (0.435)
Days off	One day off per month	0.192 ** (0.0821)
Job intensive	5-point Likert scale	−0.299 * (0.162)
Safety equipment	Having/not having safety equipment	0.141 (0.126)
Constant		0.528 (1.461)

* *p* < 0.10, ** *p* < 0.05.

## References

[1] Ministry of Employment and Labor Industrial accidents in 2018. https://www.moel.go.kr/policy/policydata/view.do;jsessionid=rJga5tGUSV7wYY9nut1nhaWRqjzQwjDBJ7sl6cLA7ArPKT0KUDHRTN9hqWhIxlKg.moel_was_outside_servlet_www1?bbs_seq=20190500060.

[2] Bureau of Labor Statistics Bureau of Labor Statistics news released. https://www.bls.gov/news.release/pdf/cfoi.pdf.

[3] Jeong B.Y. (2017). Occupational deaths and injuries by the types of street cleaning process. Int. J. Occup. Saf. Ergon..

[4] Statistics Korea Korean standard statistical classification. http://kssc.kostat.go.kr/ksscNew_web/ekssc/main/main.do#.

[5] Statistics Korea Korean standard industrial classification. http://kostat.go.kr/e_book/kssc/KSIC08/EBook.htm.

[6] KOSIS Report on the service industry survey (2012 yearly base). http://kosis.kr/2013.

[7] Choi E.-S., Sohn S.-Y., Yi K.-H. (2011). A study on types of municipal sanitation workers’ occupational accident by work type. Kor. J. Occup. Health Nurs.

[8] Kum V., Sharp A., Harnpornchai N. (2005). Improving the solid waste management in Phnom Penh city: A strategic approach. Waste Manag..

[9] Jeong B.Y., Lee S., Lee J.D. (2016). Workplace accidents and work-related illnesses of household waste collectors. Safe Health Work.

[10] Englehardt J.D., Fleming L.E., Bean J.A. (2003). Analytical predictive Bayesian assessment of occupational injury risk: Municipal solid waste collectors. Risk Anal..

[11] Poulsen O.M., Breum N.O., Ebbehøj N., Hansen Å.M., Ivens U.I., van Lelieveld D., Malmros P., Matthiasen L., Nielsen B.H., Nielsen E.M. (1995). Collection of domestic waste. Review of occupational health problems and their possible causes. Sci Total Environ..

[12] Federation of Korean Trade Unions Report of Street Cleaners Health Seminar materials for securing the right to health and preventing industrial accidents for street cleaners in 2019. http://inochong.org/storehouse/234865.

[13] Jayakrishnan T., Jeeja M.C., Bhaskar R. (2013). Occupational health problems of municipal solid waste management workers in India. Int. J. Environ. Health Eng..

[14] Kumar R., Kumar S. (2008). Musculoskeletal risk factors in cleaning occupation—A literature review. Int. J. Ind. Ergon..

[15] Ziaei M., Choobineh A., Abdoli-Eramaki M.G. (2018). Individual, physical, and organizational risk factors for musculoskeletal disorders among municipality solid waste collectors in Shiraz, Iran. Ind. Health.

[16] Lim M.S. (2010). Occupational Accidents of Sanitation Workers and the Main Causes.

[17] Faul F., Erdfelder E., Lang A.-G., Buchner A. (2007). G*Power 3: A flexible statistical power analysis program for the social, behavioral, and biomedical sciences. Behav. Res. Methods.

[18] Faul F., Erdfelder E., Buchner A., Lang A.-G. (2009). Statistical power analyses using G*Power 3.1: Tests for correlation and regression analyses. Behav. Res. Methods.

[19] IBM Corp (2013). IBM SPSS Statistics for Windows, 22.0.

[20] StataCorp (2015). Stata Statistical Software, Release 14.

[21] Abou-ElWafa H.S., El-Bestar S.F., El-Gilany A.-H., Awad E.E.-S. (2012). Musculoskeletal disorders among municipal solid waste collectors in Mansoura, Egypt: A cross-sectional study. BMJ Open.

[22] Norman I., Kretchy J., Brandford E. (2013). Neck, wrist and back pain among solid waste collectors: Case study of a Ghanaian waste management company. Public Health.

[23] Reddy E.M., Yasobant S. (2015). Musculoskeletal disorders among municipal solid waste workers in India: A cross-sectional risk assessment. J. Family Med. Prim. Care.

[24] Gonzalez J. (1995). Public Service Deficiencies and Aedes aegypti Breeding Sites in Venezuela. Bull PAHO.

[25] Ministry of Employment and Labor Industrial accidents in 2017. http://www.moel.go.kr/policy/policydata/view.do?bbs_seq=20190300037.

